# Multi-omic analysis of precocious puberty girls: pathway changes and metabolite validation

**DOI:** 10.3389/fendo.2024.1285666

**Published:** 2024-02-29

**Authors:** Fang Zhou, Jianhong Mao, Zhenzhen Jin, Li Zhu, Xiaofang Li

**Affiliations:** ^1^ Department of Pediatrics, Traditional Chinese Medicine Hospital of Zhuji, Zhuji, Zhejiang, China; ^2^ Department of Clinical Lab, Traditional Chinese Medicine Hospital of Zhuji, Zhuji, Zhejiang, China; ^3^ Department of Chinese Materia Medica, Traditional Chinese Medicine Hospital of Zhuji, Zhuji, Zhejiang, China

**Keywords:** precocious puberty, GnRH, metabolomics, transcriptomics, pathways, thymine

## Abstract

**Objective:**

Precocious puberty (PP) is a prevalent endocrine disorder affecting the physical and mental wellbeing of children. Identifying the triggering factors of PP has become a central issue. This study seeks to investigate the metabolomic and transcriptomic alterations in PP.

**Material and methods:**

First, 37 school-aged girls diagnosed with PP and 25 age-matched prepubertal control girls were recruited, and the fecal samples were collected for non-targeted metabolomic analysis to screen for differentially expressed metabolites (DEMs). Subsequently, an animal model of PP was constructed by danazol administration to neonatal female rats, and both fecal non-targeted metabolomics and serum next-generation transcriptomic sequencing were performed to screen DEMs and differentially expressed genes (DEGs) in PP. Moreover, the DEM co-existing in clinical and animal models was administrated to PP rats to explore the role of the target metabolite in PP.

**Results:**

A total of 24 DEMs in PP clinical samples and 180 DEMs and 425 DEGs in PP animal samples were identified. The Kyoto Encyclopedia of Genes and Genomes (KEGG) pathway analysis showed that these DEMs and DEGs were enriched in disease-associated pathways, including fatty acid synthesis, glycerolipid metabolism, pyrimidine metabolism, steroid hormone biosynthesis, progesterone-mediated oocyte maturation, and gonadotropin-releasing hormone (GnRH) signaling pathway, forming a tight DEM–DEG pathway regulatory network. Further DEM validation demonstrated that thymine supplementation delayed the opening of the vagina and development of PP in model rats.

**Conclusion:**

This study reveals that the metabolomic and transcriptomic changes, along with enriched pathways, are implicated in PP based on clinical and animal analyses. The findings may provide new strategies and research avenues for PP treatment.

## 1 Introduction

Precocious puberty (PP) is a prevalent endocrine disorder in pediatrics, which is manifested by accelerated growth in height and advancement and maturation of sexual characteristics and gonadal function of children at a younger age (<8 years old for girls and <9 years old for boys) (1). Epidemiological investigations have shown that the number of children with global PP continues to increase in recent years due to social and environmental impairments, especially during the global COVID-19 pandemic (2, 3). PP can be categorized into peripheral PP and central PP (CPP) according to the factors of its onset, and epidemiological data suggest that CPP manifests more frequently among PP girls. In a nationwide study in France, the incidence of CPP was 2.68 per 10,000 in girls and 0.24 per 10,000 in boys (4). A population-based cohort study among children in Denmark reported that the incidence of PP girls ranges from 2.6 per 10,000 to 14.6 per 10,000 and that of PP boys from 0.1 per 10,000 to 2.1 per 10,000 from 1998 to 2017 (2). CPP is gonadotropin-releasing hormone (GnRH) dependent, instigated by premature activation of the hypothalamic–pituitary–gonadal (HPG) axis, promoting the secretion of GnRH, which triggers the development of gonads and the secretion of sex hormones, resulting in the development of internal and external genitalia and the presentation of secondary sexual characteristics (5). CPP can also lead to premature skeletal development and closure, whereas hindrance to height development negatively affects the physical and mental health of children. A study also indicated that children with PP may have increased risks of hypertension, diabetes, obesity, and infertility in their adulthood (6). Therefore, the identification of puberty-initiating factors has emerged as a vital issue in the studies of child sexual development.

It is widely recognized that the mechanisms and timing of puberty are driven by the interaction of a series of complex factors, which can be mainly categorized into endogenous and exogenous causes (7). Endogenous factors primarily refer to the patient’s endocrine abnormalities. Exogenous factors, on the other hand, include environmental elements, lifestyle, nutritional intake, obesity, and certain diseases. Obesity and environmental pollutants such as endocrine-disrupting chemicals (EDCs) are now acknowledged as the main triggers of CPP (8, 9). Adipocytes contain precursors of estrogen, which can be converted to estrogen to stimulate gonadal maturation. The level of estrogen is significantly increased when excess fat is accumulated in the body (10). Data suggest that the GnRH-neuronal network is highly sensitive to EDCs during development at puberty. EDCs regulate the activation of GnRH and the production of gonadal steroids, thereby prematurely initiating puberty via the HPG axis (11, 12). Despite the rising tide of studies attempting to explain the pathogenesis of PP, the affecting factors and regulatory networks involved in it remain elusive.

Recently, with the rapid development of multiple high-throughput screening technologies, the integrated analysis of multi-omics has been widely used in medical research, which provides great convenience in deciphering the pathogenesis of complex diseases, screening biomarkers, and providing therapeutic strategies (13, 14). Metabolomics, proteomics, transcriptomics, lipidomics, microbiome single-cell transcriptomics, and other technologies reveal the interactions of multiple substances in organisms and the impacts on phenotypes and traits from different perspectives. In this study, PP girls and age-matched prepubertal girls were recruited through a clinical trial, and fecal samples were collected for non-targeted metabolomic analysis to screen differentially expressed metabolites (DEMs). Subsequently, an animal model of PP was constructed with danazol, and fecal non-targeted metabolomics was performed to screen for DEMs. To provide further insight into the possible role of DEMs in PP development, co-existing DEMs in clinical and animal models were screened and validated in a PP animal model to explore the roles of the target metabolite in PP. By performing multi-omics in clinical and animal samples of PP, this study seeks to provide potential biomarkers that could contribute to the diagnosis and treatment of PP.

## 2 Materials and methods

### 2.1 Clinical subjects

The study recruited 37 girls diagnosed with PP and 25 age-matched prepubertal girls as controls from pediatric endocrinology clinics of the Traditional Chinese Medicine Hospital of Zhuji from 2019 to 2021. *All girls in both groups voluntarily joined this study with informed consent, and this study was approved by the ethics committee of the Zhuji City Hospital of Traditional Chinese Medicine.* The inclusion criteria of PP referred to the diagnostic guidelines of PP (15), whereas the exclusion criteria are outlined as follows: 1) participators with other serious diseases and taking medication for a long time or within the last 3 days, 2) incomplete information, and 3) not signing the informed consent or cooperating. The clinical characteristics, including age, bone age, height, weight, and body mass index (BMI), were collected. The levels of sex hormone and steroid hormones were detected before grouping, including follicle-stimulating hormone (FSH), luteinizing hormone (LH), E2 (E_2_), progesterone, androstendione, and dehydroepiandrosterone. To avoid subjectivity in sample selection that might affect data analysis, the basic clinical characteristics of the two groups have no significant differences by Student’s *t*-test.

### 2.2 Clinical metabolomic analysis

Fresh fecal samples from each participant (37 PP and 25 control girls) were collected for untargeted metabolomic analysis by Metabo-Profile. Briefly, an Agilent 7890B gas chromatograph and a time-of-flight mass spectrometry system (GC-TOF/MS, Pegasus HT, Leco Corp., USA) were used for untargeted metabolomic analysis. The chromatographic column was an Rxi-5ms capillary column (inner diameter 30 m × 250 μm, film thickness 0.25 μm; Restek Corporation, USA). Helium was maintained at a constant flow rate of 1.0 mL/min. The temperature for both injection and transfer interface was set to 270°C. Measurements were performed in full scan mode (m/z 50–500) using electron impact ionization (70 eV). Quality control procedures were performed through sample preparation and analytical testing. The raw data generated by GC-TOF/MS were processed for metabolite characterization and quantification using the XploreMET software (Metabo-Profile, Shanghai, China). Statistical analysis was performed using Student’s *t*-test to identify DEMs, and statistical differences between the two groups were defined as *p* < 0.05 and |log_2FoldChange_| > 1.

### 2.3 Animal experimental design

Sprague-Dawley female offspring rats (3 days old) with their mothers were purchased from Shanghai SLAC Laboratory Animal Co., Ltd. (Certification No. SCXK(Hu)2017-0005). In this study, two successive animal experiments were designed. For the first experiment, the rats were randomly divided into the control group and PP group. The PP animal model was established according to a previous publication (16). Briefly, at 5 days old, female offspring Sprague-Dawley (SD) rats received a subcutaneous injection of a single dose of 300 µg danazol (D132177, Aladdin). Danazol was dissolved in 30 µL of solvent (propylene glycol: ethanol, 1:1, v/v). Other rats receiving the solvent alone acted as the control group. After danazol injection, the time of vaginal opening was observed daily for each rat and photographed. For the second experiment, the rats were randomly divided into two groups, the PP group and thymine-treated group. The PP model group was established in the same way as the abovementioned described method. The thymine-treated group was supplemented with thymine (gavage, 1 g/kg/day, S18065, Shanghai Yuanye Bio-Technology Co., Ltd.) after PP modeling (17). Each group had 12 rats; 6 rats were used to observe the time of vaginal opening, and the remaining 6 rats were used for sample collection. The animal experimental protocol was in accordance with *the Guidelines for the Care and Use of Laboratory Animals of the National Institutes of Health*; the protocol was approved by *the Animal Experimentation Ethics Committee of Zhejiang Eyong Pharmaceutical Research and Development Center* (SYXK(Zhe)2021-0033).

### 2.4 Sample collection

After the vagina of the last rat in the model group was opened, six rats in each group were weighed and then anesthetized with 2% isoflurane. Blood samples were collected from the abdominal aorta and centrifugated at 3,000 rpm for 15 min to obtain supernatant serum samples. Then, the rats were euthanized with CO_2_; the uterus and ovary, hypothalamus, and hypophysis were isolated, photographed, and weighed. The organ index was calculated according to the following formula: organ index (%, mg/g) = 100% × organ weight (mg)/whole body weight (g). Samples were stored at −80°C until further analysis.

### 2.5 ELISA

Serum samples were used for hormone level detection. The levels of FSH, LH, E_2_ (MM-70867R2, MM-0624R2, MM-0575R2, MEIMIAN), and leptin (PL698, Beyotime) in serum were detected with the specific ELISA kits according to the manufacturer’s instructions. The OD value was detected at 450 nm by an ELISA reader (CMaxPlus, MD).

### 2.6 HE and immunohistochemical staining

The uterus and ovarian tissues were fixed with 4% paraformaldehyde, embedded with paraffin, and sliced into 5-mm sections. The uterus and ovarian sections were stained with hematoxylin and eosin solution successively. After being sealed with neutral gum, the sections were observed under a light microscope (Nikon Eclipse Ci-L, Nikon). The fixed ovarian tissue sections were deparaffinized with xylene, hydrated with ethanol, and placed in antigen repair solution for antigen repair, followed by incubation with primary antibody anti-GnRH (Bioss, bs-10369R), washing with phosphate-buffered saline (PBS), and then incubation with secondary antibody immunoglobulin G (IgG) H&L [horseradish peroxidase (HRP)] (Abcam, ab97080). After washing with PBS, 3,3′-diaminobenzidine (DAB) solution was used to develop the color, and hematoxylin was used to re-stain the slices. Finally, the dehydrated and sealed sections were placed under a microscope for observation (Nikon DS-U3, Nikon).

### 2.7 qRT-PCR

The messenger RNA (mRNA) levels of GnRH, GnRH receptor (GnRHR), netrin-1, and KISS1 in hypothalamus tissues and those of luteinizing hormone receptor (LHR) and follicle-stimulating hormone receptor (FSHR) in uterine tissues were determined by quantitative reverse transcription PCR (qRT-PCR), respectively. The total RNA was extracted using EZ-10 Total RNA Mini-Preps Kit (B618583-0100, Sangon Biotech). The extracted RNA was reverse-transcribed into complementary DNA (cDNA) by the HiFiScript cDNA synthesis kit (CW2569, CWBIO), and qRT-PCR analysis was performed using a Roche LightCycler^®^ 96 qRT-PCR instrument according to the manufacturer’s instructions. The primer sequences of these detected genes are listed in **Table 1**. The relative expression of target genes was determined using the 2^−ΔΔCq^ method and normalized to GAPDH.

**Table 1:** Primer sequences.

Gene	Forward primer (5′–3′)	Reverse primer (5′–3′)
Rat GnRH	CCGCTGTTGTTCTGTTGACT	GCAGATCCCTAAGAGGTGAA
Rat GnRHR	TCACTCAGCCCTTAGCTGTCC	GAAGGCTTCATGCCACCATTG
Rat netrin-1	AGAGTTTGTGGATCCGTTCG	TTCTTGCACTTGCCCTTCTT
Rat KISS1	CAATGGTCTGAACTGCCCAC	CACAGGTGCCATTTTTGCCA
Rat FSHR	ATTCTTGGGCACGGGATCTG	GTGGGTAGCGGATGAGGTTT
Rat LHR	CTTCACAGCTGCAGTCCCGA	CTCGGTGGTATGGGCTGTTG
Rat GAPDH	GATGGTGAAGGTCGGTGTGA	TGAACTTGCCGTGGGTAGAG

### 2.8 Western blot

The protein expressions of phospho-carbamoyl-phosphate synthetase-aspartate carbamoyl transferase-dihydro-orotase (P-CAD), CAD, and carbamoyl phosphate synthetase 2 (CPS2) in ovarian tissues were detected by Western blotting. Briefly, after the total protein concentration of ovarian tissue was determined with a BCA kit, a 20 µg total target protein sample was electrophoretically separated by 5% sodium dodecyl sulfate–polyacrylamide gel electrophoresis (SDS-PAGE) gel and subsequently transferred to a polyvinylidene fluoride (PVDF) membrane. The membrane was closed with 5% skimmed milk powder at the end of the transfer, followed by the addition of primary antibody dilution, including P-CAD (AF4415, Affinity), CAD (AF4715, Affinity), CPS2 (sc-376072, Santa Cruz), and GAPDH (10494-1-AP, Proteintech) for overnight incubation at 4°C. After that, the secondary antibody IgG H&L (HRP, 7074, CST) was added for further incubation. After washing the membrane, the blotting was visualized with an enhanced chemiluminescence (ECL) reagent; the blot was photographed using chemi-capture software and analyzed by the ImageJ software.

### 2.9 Metabolomic analysis

Fresh fecal samples from each rat were collected for untargeted metabolomic analysis by PANOMIX Biomedical Tech Co., Ltd. Briefly, liquid chromatography analysis was performed with a Vanquish ultrahigh-performance liquid chromatography (UHPLC) system (Thermo Fisher Scientific) with an ACQUITY UPLC^®^ HSS T3 column (2.1 mm × 100 mm, 1.8 µm, Waters), at a flow rate of 0.3 mL/min and an injection volume of 2 μL. The mass spectrometric detection of metabolites was performed on a Q Exactive instrument (Thermo Fisher Scientific, USA) with an electrospray ionization (ESI) ion source. The capillary temperature was set at 325°C, the MS scan range was m/z 100–1,000, the resolving power was 70,000 full width at half maximum (FWHM), and the MS/MS resolving power was 17,500 FWHM with a collision energy of 30 eV. Quality control procedures were performed through sample preparation and analytical testing. The raw data were processed by the R XCMS software package for metabolite characterization and quantification. Student’s *t*-test and orthogonal partial least squares–discriminant analysis (OPLS-DA) analysis were both performed to identify DEMs, and statistical differences between the two groups were defined as *p* < 0.05 and Variable Importance for the Projection (VIP) > 1.

### 2.10 Next-generation transcriptomic sequencing

Serum samples were collected for next-generation sequencing by PANOMIX Biomedical Tech Co., Ltd. Following total RNA extraction from the samples, mRNA purification, and cDNA synthesis, the libraries were subjected to quality control with an Agilent 2100 Bioanalyzer, and the total concentration of the libraries was detected by fluorescence quantification. Subsequently, the samples were uploaded for next-generation sequencing based on the Illumina sequencing platform and the raw data were obtained. After the raw data were filtered and quality assessed, the filtered reads were statistically analyzed to the reference genome using the upgraded version of the HISAT2 software, and the read count value obtained was used as the raw expression of the gene. Based on this, the differentially expressed genes (DEGs) were screened, and the criteria were |log_2_ fold change| > 1 and *p* < 0.05. After that, Kyoto Encyclopedia of Genes and Genomes (KEGG) enrichment analysis of the DEGs was performed based on the Database for Annotation, Visualization, and Integrated Discovery (DAVID database), and transcription factor analysis was performed based on the Animal Transcription Factor Database (AnimalTFDB).

### 2.11 Statistical analysis

The studies were analyzed using SPSS 20.0, and the values are displayed as means ± SD. Comparison of two groups was performed by Student’s *t*-test. *p* < 0.05 was considered statistically significant.

## 3 Results

### 3.1 Clinical characteristics and hormone levels of the PP girls

The clinical characteristics of two groups are summarized in **Table 2**. The bone age of girls in the PP group was older than that in the control group; the weight, height, and BMI were also significantly higher than those in the age-matched prepubertal girls (*p* < 0.01). More importantly, the serum levels of sex hormones and steroid hormones showed that E2, androstendione, and dehydroepiandrosterone in the PP group were higher than those in the prepubertal group girls, respectively (*p* < 0.05).

**Table 2:** Clinical information on the recruited subjects.

Clinical characteristics	Control group	PP group	*P*-value
Age (years)	6.64 ± 0.50	6.76 ± 0.44	0.449
Bone age (years)	7.25 ± 0.96	8.00 ± 1.15	0.044 < 0.05
Weight (kg)	23.46 ± 4.20	27 ± 3.06	<0.01
Height (cm)	122.78 ± 4.64	126.56 ± 5.04	0.023 < 0.05
BMI (kg/m2)	15.49 ± 1.98	16.83 ± 1.64	0.029 < 0.05
FSH	1.63 ± 1.01	2.04 ± 1.83	0.456
LH	0.200 ± 0.015	0.292 ± 0.370	0.381
E2	130.0 ± 109.2	194.54 ± 157.60	<0.01
Progesterone	0.235 ± 0.315	0.622 ± 0.832	0.104
Androstendione	0.108 ± 0.068	0.294 ± 0.276	0.018 < 0.05
Dehydroepiandrosterone	0.418 ± 0.303	0.799 ± 0.548	0.022 < 0.05

FSH, follicle-stimulating hormone; LH, luteinizing hormone; E2, estradiol.

### 3.2 Metabolomic changes in the feces of PP girls

**Figure 1:**
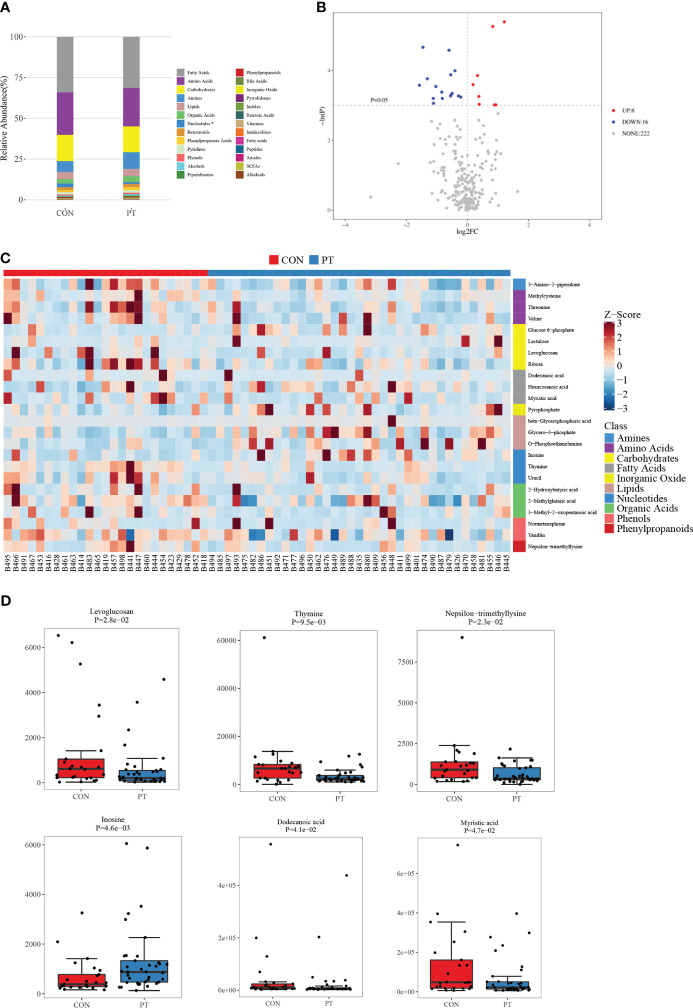
Metabolomic analysis of the feces of 37 precocious puberty (PP) girls and 25 age-matched prepubertal girls. **(A)** Relative abundance of each metabolite class. **(B)** Volcano plot of differentially expressed metabolites (DEMs). **(C)** Heat map of 24 DEMs in PP and prepubertal samples. **(D)** Boxplot of the top six fold-changed DEMs. PP, precocious puberty; DEMs, differentially expressed metabolites.

**Figure 2:**
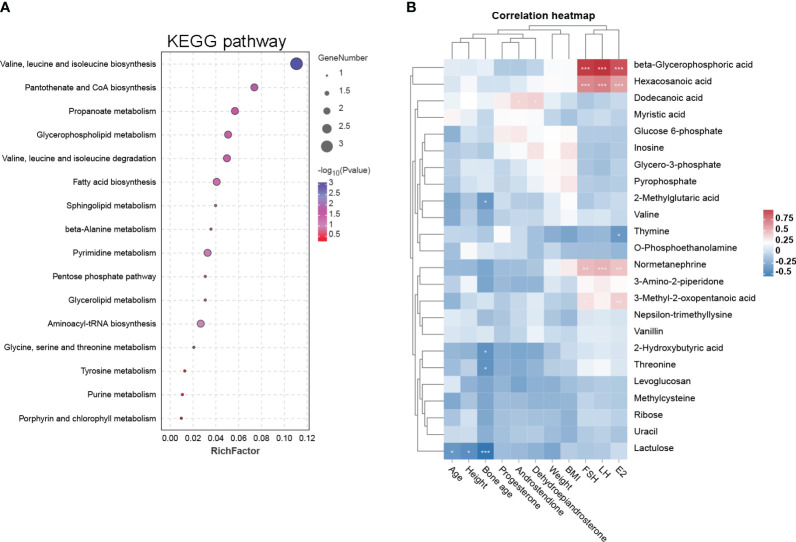
KEGG pathway and clinical correlation analysis of DEMs. **(A)** The 17 DEM-enriched KEGG pathways are represented by a bubble diagram. The size of the bubble represents the number of the enriched DEMs, and the color represents −log 10 (P-value). **(B)** Correlation heat map of DEMs and clinical characteristics in PP and prepubertal girls. Blue represents negative correlation, while red represents positive correlation; *p < 0.05, **p < 0.01, ***p < 0.001.

Fecal samples from PP girls and age-matched prepubertal girls were analyzed by the metabolomic method. The relative abundance of the detected metabolites is presented in **Figure 1A**; it could be seen that most metabolites belong to fatty acids, amino acids, carbohydrates, amines, and lipids. Based on the criteria of *p* < 0.05 and ∣log_2_ fold change∣ ≥ 0, 8 up- and 16 downregulated metabolites were identified from the feces of PP girls compared to that of the control prepubertal girls (**Figure 1B**, **Table 3**). As a result, these 24 metabolites were identified as the DEMs in PP girls; they were fatty acids, lipids, carbohydrates, amino acids, organic acids, and phenols. The heat map for the level of these DEMs in two group samples is shown in **Figure 1C**. The levels of the DEMs with the top ∣log_2_ fold change∣ value in two groups were draw by boxplot, including levoglucosan, thymine, nepsilon-trimethyllysine, inosine, dodecanoic acid, and myristic acid (**Figure 1D**). KEGG pathway analysis also showed that these DEMs were enriched in fatty acid biosynthesis, glycerophospholipid metabolism, glycerolipid metabolism, pyrimidine metabolism, steroid biosynthesis, ovarian steroidogenesis, etc. (**Figure 2A**).

**Table 3:** Differential metabolites identified from the feces of PP girls.

No.	Metabolite	Class	Formula	KEGG	P-value	log_2_FC	VIP
1	2-Hydroxybutyric acid	Organic acids	C4H8O3	C05984	0.0292	−1.037	0.6713
2	2-Methylglutaric acid	Organic acids	C6H10O4	NA	0.0186	−0.412	0.2524
3	3-Amino-2-piperidone	Amines	C5H10N2O	NA	0.0381	−0.2969	1.6678
4	3-Methyl-2-oxopentanoic acid	Organic acids	C6H10O3	C00671	0.0343	−0.8422	1.1534
5	beta-Glycerophosphoric acid	Lipids	C3H9O6P	C02979	0.0388	0.3727	0.9327
6	Hexacosanoic acid	Fatty acids	C26H52O2	NA	0.0485	0.3827	0.1853
7	Dodecanoic acid	Fatty acids	C12H24O2	C02679	0.041	−1.1197	1.2725
8	Glucose 6-phosphate	Carbohydrates	C6H13O9P	C00092	0.0493	0.8858	1.7846
9	Glycero-3-phosphate	Lipids	C3H9O6P	C00093	0.0053	0.8301	2.1649
10	Inosine	Nucleotides	C10H12N4O5	C00294	0.0046	1.2049	1.4595
11	Valine	Amino acids	C5H11NO2	C00183	0.0104	−0.6053	1.0679
12	Lactulose	Carbohydrates	C12H22O11	C07064	0.0493	0.9302	1.4973
13	Levoglucosan	Carbohydrates	C6H10O5	NA	0.0281	−1.5756	1.0159
14	Methylcysteine	Amino acids	C4H9NO2S	NA	0.0209	−0.549	1.4087
15	Myristic acid	Fatty acids	C14H28O2	C06424	0.0471	−1.1119	1.9383
16	Nepsilon-trimethyllysine	Phenylpropanoids	C27H30O15	NA	0.0234	−1.3156	1.7291
17	Normetanephrine	Phenols	C9H13NO3	C05589	0.041	−0.8242	1.2408
18	O-Phosphoethanolamine	Lipids	C2H8NO4P	C00346	0.0276	0.1811	1.8916
19	Pyrophosphate	Inorganic oxide	H4O7P2	C00013	0.0213	0.3267	2.3957
20	Ribose	Carbohydrates	C5H10O5	C00121	0.0355	−0.5031	2.0323
21	Threonine	Amino acids	C4H9NO3	C00188	0.0381	−0.5522	1.6654
22	Thymine	Nucleotides	C5H6N2O2	C00178	0.0095	−1.4592	1.9692
23	Uracil	Nucleotides	C4H4N2O2	C00106	0.0368	−0.5313	1.8968
24	Vanillin	Phenols	C8H8O3	C00755	0.0394	−0.2181	1.4958

### 3.3 Correlations between differential metabolites and clinical characteristics

Based on these 24 DEMs, we further analyzed their clinical relevance. Correlations between these DEMs and clinical characteristics are represented by a heat map (**Figure 2B**). The data are explained as follows: (1) beta-glycerophosphoric acid, hexacosanoic acid, normetanephrine, and 3-methyl-2-oxopentanoic acid have significant positive correlations with FSH, LH and E2; (2) dodecanoic acid has positive correlations with dehydroepiandrosterone and androstendione; and (3) thymine has a significant negative correlation with E2.

### 3.4 Successful establishment of the PP model by danazol in neonatal female rats

**Figure 3:**
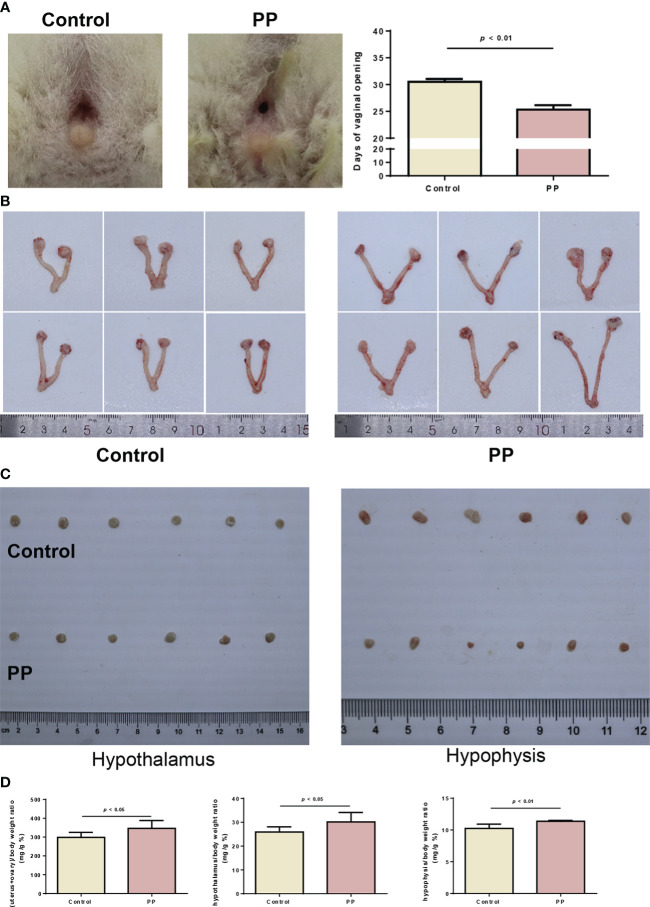
Danazol advanced the time of vaginal opening and promoted sex organ development in neonatal female rats (n = 6). **(A)** The representative images and days of vaginal opening in two groups of female rats. **(B)** The bilateral ovaries and uterus from the two rat groups were obtained and photographed. **(C)** The hypothalamus and hypophysis from the two rat groups were obtained and photographed. **(D)** The organ indexes of the uterus and ovary, hypothalamus, and hypophysis were calculated.

**Figure 4:**
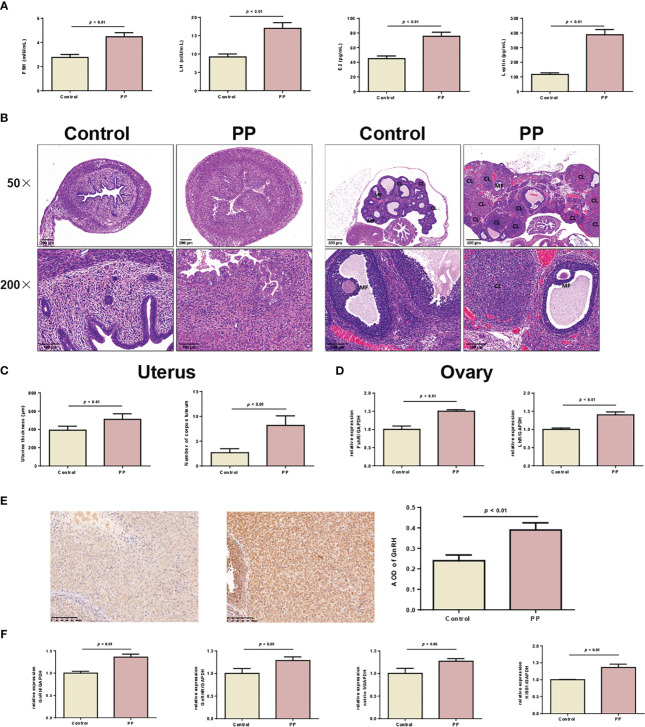
Danazol promoted the maturation of follicles and thickening of the uterine wall in neonatal female rats. **(A)** Serum levels of FSH, LH, E2, and leptin in the two rat groups were detected by ELISA (n = 6). **(B)** HE staining of the uterus (×50, scale bar = 200 mm; ×200, scale bar = 100 mm) and ovary of two rat groups (×50, scale bar = 500 mm; ×200, scale bar = 100 mm) (n = 6). CL, corpus luteum; MF, mature follicle. **(C)** The thickening of the uterine wall and the number of corpora lutea were calculated. **(D)** The mRNA expression of FSHR and LHR in uterine tissues were detected by qRT-PCR (n = 3). **(E)** The expression of GnRH in ovarian tissues was detected by immunohistochemical staining (n = 6). **(F)** The mRNA levels of GnRH, GnRHR, netrin-1, and KISS1 in hypothalamus tissues were detected by qRT-PCR (n = 6).

Based on the results of the metabolomic changes in clinical PP girls, we further induced PP by danazol in female SD rats. By monitoring the vaginal opening time, we found that danazol administration significantly shortened the time of vaginal opening of female rats in the PP model group (**Figure 3A**, 25.3 days vs. 30.5 days, *p* < 0.01). Furthermore, the organ indexes of uterus, ovary, hypothalamus, and hypophysis were increased significantly in the PP group after danazol administration (**Figures 3B–D**, p < 0.05). Furthermore, significantly increased sex hormone levels, follicle maturation, and uterine wall thickening were also found in danazol-treated rats (**Figures 4A–D**, *p* < 0.01). The mRNA expressions of FSHR and LHR in the ovary and GnRH, GnRHR, netrin-1, and KISS1 in the hypothalamus were also increased (**Figures 4E, F**, *p* < 0.01).

### 3.5 Metabolomic changes in the feces of PP rats

**Figure 5:**
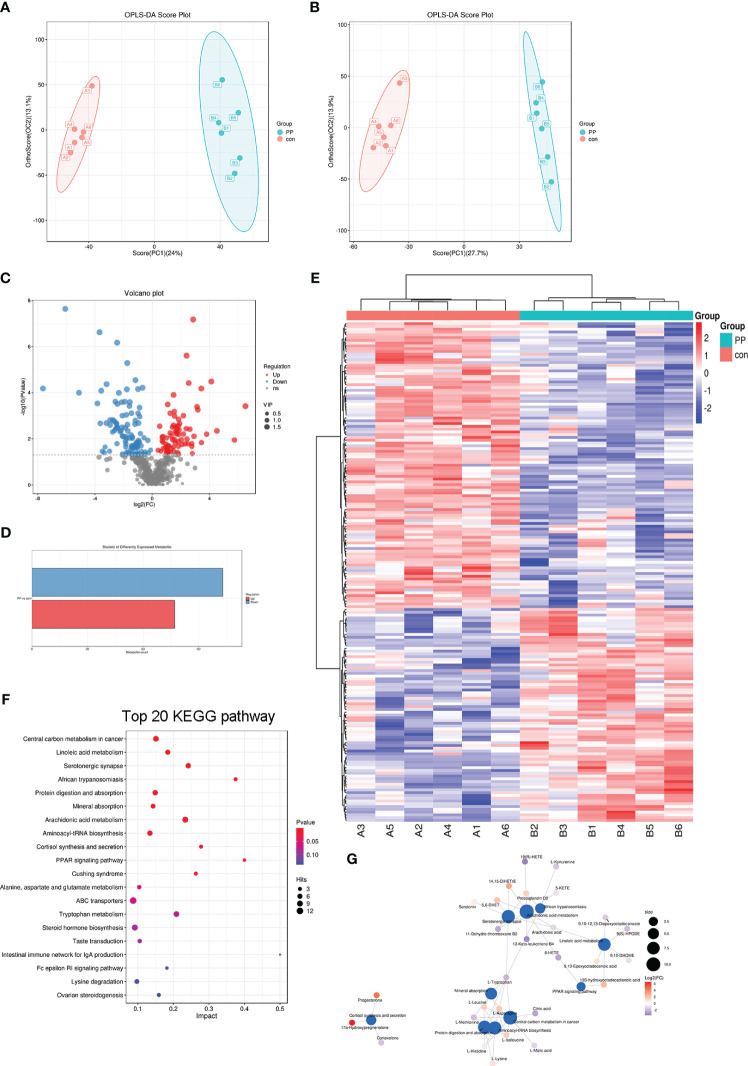
Metabolomic analysis of the feces of PP and control rats. **(A)** OPLS-DA score in positive mode. **(B)** OPLS-DA score in negative mode. **(C)** Volcano plot of DEMs. **(D)** Count statistics of DEMs; there are 77 upregulated and 103 downregulated DEMs between the two group rats. **(E)** Heat map of 180 DEMs in PP and control samples. A1–A6 represent control samples; B1–B6 represent PP samples. **(F)** Bubble diagram of the top 20 DEM-enriched KEGG pathways. **(G)** KEGG pathway network.

The feces of PP and control rats were collected for metabolomic analysis. The OPLS-DA model showed clear discrimination between the two groups (**Figures 5A, B**). By OPLS-DA model analysis, a total of 77 up- and 103 downregulated metabolites were identified from the feces of PP rats compared to those in the feces of the control rats (**Figures 5C, D**, **Supplementary Table 1**). There were two DEMs that overlapped in clinical and animal fecal samples, thymine and inosine. The expression heat map of these DEMs in two group samples is shown in **Figure 5E**. KEGG pathway analysis showed that these 180 DEMs were enriched in the pathways of cortisol synthesis and secretion, arachidonic acid metabolism, steroid hormone biosynthesis, ovarian steroidogenesis, progesterone-mediated oocyte maturation, GnRH signaling pathway, biosynthesis of unsaturated fatty acids, glycerolipid metabolism, pyrimidine metabolism, etc. (**Figure 5F**). Meanwhile, these pathways formed a tight connection network (**Figure 5G**).

### 3.6 Transcriptomic changes in the serum of PP rats

**Figure 6:**
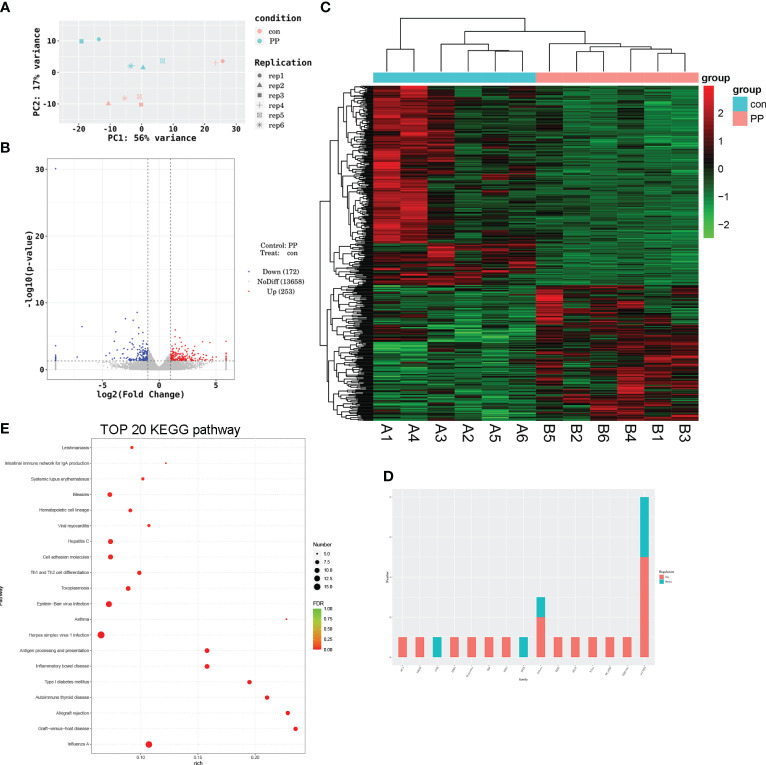
Transcriptomic analysis of the serum of PP and control rats. **(A)** Principal component analysis of RNA-sequencing samples. **(B)** Volcano plot of differentially expressed genes (DEGs) involved in 253 upregulated and 172 downregulated DEGs. **(C)** Heat map of DEGs in PP and control samples. **(D)** Transcription factor analysis of DEGs. **(E)** Bubble diagram of the top 20 DEG-enriched KEGG pathways.

The sera of PP and control rats were collected for RNA sequencing. The principal component analysis (PCA) model showed good discrimination between the two groups (**Figure 6A**), and a total of 425 DEGs were identified from the PP rats when compared to the those of the control rat, including 253 upregulated and 172 downregulated DEGs (**Figure 6B**). The expression heat map of these DEGs in two group samples is shown in **Figure 6C**. Transcription factor analysis showed that some of these DEGs are transcription factors and belong to different transcription factor families. **Figure 6D** calculates the number of DEGs in transcription factor families, 5 upregulated DEGs (Zfp316, Zfp54, Zfp646, Rest, and LOC103690114) and 3 downregulated DEGs (LOC103690028, Zfp691, and Zfp394) belong to the zf-C2H2 family, the upregulated DEG Thrb belongs to the THR-like family, the upregulated DEG Stat1 belongs to the STAT family, the upregulated DEG Smad1 belongs to the MH1 family, etc. These DEGs were enriched in multiple pathways; the top 20 are presented in **Figure 6E**.

### 3.7 Integrative analysis of metabolomic and transcriptomic data

**Figure 7:**
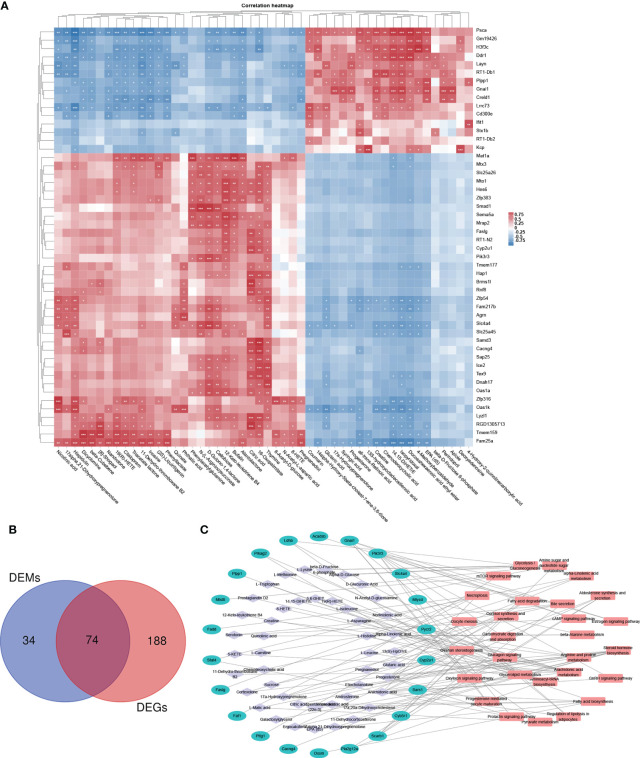
Integrative analysis of metabolomic and transcriptomic data. **(A)** Correlation heat map of the top 50 DEMs and DEGs. **(B)** Venn diagram of the overlapping KEGG pathways between metabolomic and transcriptomic analyses, DEMs- and DEGs-enriched in 74 identical pathways. **(C)** DEM–DEG KEGG pathway network. Among the 74 pathways, hub KEGG pathways that are DEM and DEG co-enriched are represented by a network. *p < 0.05, **p < 0.01, ***p < 0.001.

By integrating metabolomic and transcriptomic data, correlation analysis of the top 50 DEMS and DEGs was performed (**Figure 7A**). The correlation heat map demonstrated significant positive or negative correlations between DEMS and DEGs in fecal samples of PP rats. In addition, based on the analyzed KEGG pathways, DEMs- and DEGs-enriched in 74 identical pathways, including ovarian steroidogenesis, progesterone-mediated oocyte maturation, glycerolipid metabolism, fatty acid biosynthesis/degradation, arachidonic acid metabolism, and GnRH signaling pathway (**Figure 7B**). Among the 74 pathways, DEM- and DEG-co-enriched KEGG pathways are represented by an interrelated DEM–DEG KEGG pathway network (**Figure 7C**).

### 3.8 Thymine administration slowed down PP development in model rats

**Figure 8:**
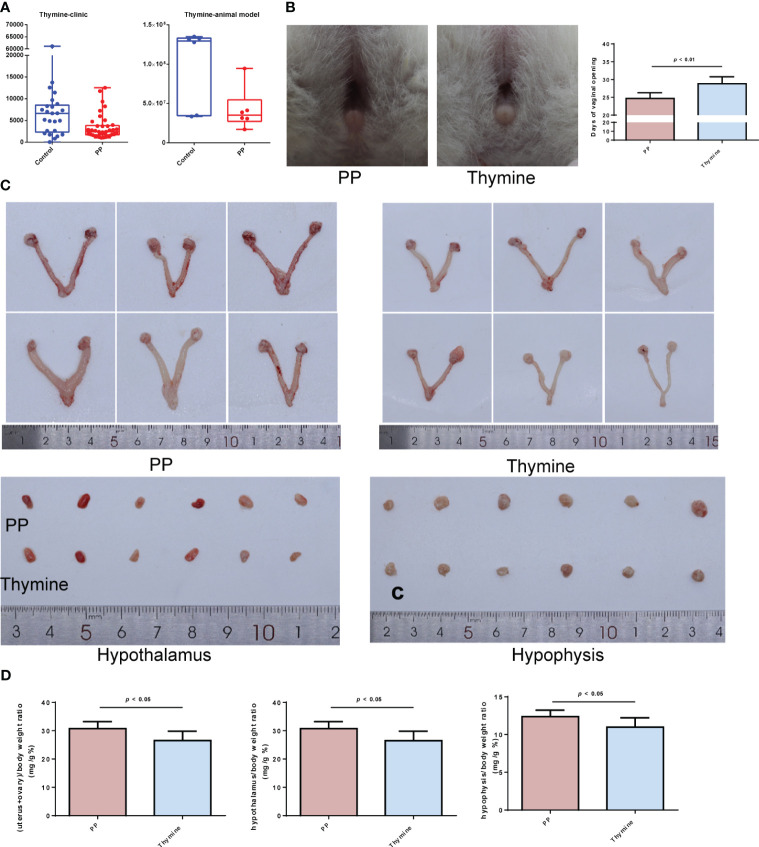
Thymine administration slows down the time of vaginal opening and reduces sex organ development in PP model rats. **(A)** The levels of thymine in human and animal fecal samples. **(B)** The representative images and days of vaginal opening in the two groups of female rats (n = 6). **(C)** The bilateral ovaries and uterus, hypothalamus, and hypophysis from the two rat groups were obtained and photographed (n = 6). **(D)** The organ indexes of the uterus and ovary, hypothalamus, and hypophysis were calculated (n = 6).

**Figure 9:**
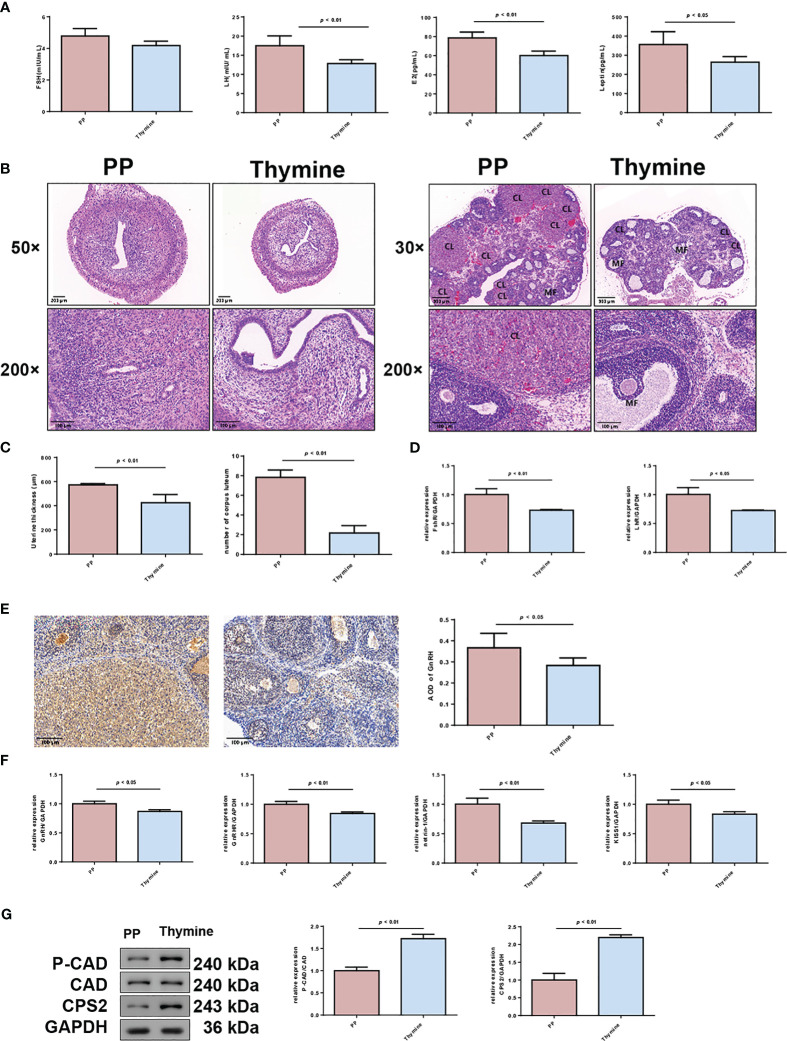
Thymine administration delays the maturation of follicles and thickening of the uterine wall in PP model rats. **(A)** Serum levels of FSH, LH, E_2_, and leptin in two rat groups were detected by ELISA (n = 6). **(B)** HE staining of the uterus (×50, scale bar = 200 mm; ×200, scale bar = 100 mm) and ovary of two rat groups (×50, scale bar = 500 mm; ×200, scale bar = 100 mm) (n = 6). CL: corpus luteum; MF: mature follicle. **(C)** The thickening of the uterine wall and number of corpora lutea were calculated. **(D)** The mRNA expressions of FSHR and LHR in uterine tissues were detected by qRT-PCR. n = 3. **(E)** The expressions of GnRH in ovarian tissues were detected by immunohistochemical staining (n = 6). **(F)** The mRNA levels of GnRH, GnRHR, netrin-1, and KISS1 in hypothalamus tissues were detected by qRT-PCR (n = 3). **(G)** The protein expressions of p-CAD/CAD and CPS 2 were detected by Western blotting (n = 3).

According to the result of clinical and animal metabolomic analysis, thymine was found as a co-downregulated DEM in PP clinical and animal model samples (**Figure 8A**); thus, we further explored its roles in PP. By thymine supplementation in PP model rats, the time of vaginal opening was statistically delayed (**Figure 8B**, *p* < 0.01). Meanwhile, the organ indexes of the uterus and ovary, hypothalamus, and hypophysis were decreased in the PP group after thymine administration (**Figures 8C, D**, *p* < 0.05). In addition, sex hormone level detection showed that the increased levels of LH, E2, and leptin were suppressed in the thymine-administrated group (**Figure 9A**, *p* < 0.05). HE staining of the uterus and ovary showed that thymine supplementation statistically reduced the endometrial thickness and the number of corpora lutea when compared with those of the PP model group (**Figures 9B, C**, *p* < 0.01). The upregulated expression of FSHR and LHR in ovarian tissues and those of GnRH, GnRHR, netrin-1, and KISS1 in the hypothalamus were significantly decreased in the thymine-administrated group (**Figures 9D–F**, *p* < 0.05). Furthermore, the proteins involved in thymine synthesis were also detected. The protein expressions of p-CAD/CAD and CPS2 regulating thymine synthesis were increased in ovarian tissues of the thymine-administrated group when compared to those in PP group rats (**Figure 9G**, *p* < 0.01).

## 4 Discussion

PP is a common endocrine disorder in pediatrics, particularly prevalent in girls, and has adverse effects on the physical and mental wellbeing of children (1). Therefore, discerning the underlying triggers of puberty has become a central issue in the research of pediatric sexual development. As multi-omic studies are able to explain complex diseases more comprehensively from different perspectives, they have recently been employed in studies related to PP, yielding some meaningful findings. Li et al. performed analysis of proteomics and metabolomics in girls with CPP and found some key differential proteins and metabolites, including MMP9, SPP1, CDC42, POSTN, COL1A1, BMP1, and phosphocholine (16:1(9Z)/16:1(9Z)) (18). Huang et al. performed 16S ribosomal RNA (rRNA) sequencing and untargeted metabolomic profiling of CPP patients; it was revealed that nitric oxide synthesis and *Streptococcus* were closely associated with CPP (19). Different from these studies, in this study, we conducted metabolomic analysis of fecal samples of clinical PP girls and animal models and further validated the role of significantly changed metabolites at the animal level.

By recruiting PP and normal girls, 24 differential metabolites were identified from feces in this study; the upregulated DEMs were carbohydrate (glucose 6-phosphate, lactulose), fatty acid (hexacosanoic acid), and lipid classes (glycero-3-phosphate, O-phosphoethanolamine, beta-glycerophosphoric acid), while the downregulated DEMs mainly included amino acids (threonine, valine, methylcysteine), organic acids (3-methyl-2-oxopentanoic acid, 2-hydroxybutyric acid), phenols (normetanephrine, vanillin), and nucleotides (thymine, uracil). In overweight or obese children, urinary metabolomic analysis also identified glucose 6-phosphate as the DEM, and it was correlated with the concentration of phthalate, which is an obesogen contributing to overweight and obesity (20). The relationship between obesity and PP is well recognized. Liu et al. reported a case–control study recruiting 846 CPP children and 1,650 healthy control subjects, and they found that overweight and obesity were significantly associated with increased odds of CPP among girls (overweight: p = 0.02; obesity: p = 0.03) (21). In another school population-based study, researchers found that the prevalence of PP among overweight (27.94%) and obese girl (48.00%) was higher than that of normal-weight girls (8.73%) (p < 0.05) (8). In Pereira et al.’s study, they performed a longitudinal study in which 494 boys and found that obesity at 4 to 7 years and the childhood mean BMI standard deviation score were significantly associated with PP (22). High accumulation of hexacosanoic acid as a lipid biomarker of dementia was found in plasma (23). Wang et al. performed untargeted metabolomic analysis in PP children and found that the pathways of threonine metabolism was perturbed in boys (24). Li et al. also reported that their identified DEMs in the sera of CPP girls were mainly involved in lipid and glycerophospholipid metabolism (18). More importantly, endocrine disruptors were reported to disrupt puberty, hormone sensitivity, and metabolism of glucose, fatty acids, and triglycerides in neonatal mice (25). Consistently, in our studies, pathway analysis also showed that these DEMs were involved in fatty acid biosynthesis, glycerophospholipid metabolism, glycerolipid metabolism, pyrimidine metabolism, steroid biosynthesis, ovarian steroidogenesis, etc. More importantly, several of these DEMs were significantly correlated with the clinical sex hormone and steroid hormone levels, including beta-glycerophosphoric acid, hexacosanoic acid, normetanephrine and 3-methyl-2-oxopentanoic acid, dodecanoic acid, and thymine. This indicates that the direct associations of these DEMs change with PP, and it provides an important reference for further exploration of these DEMs in the pathogenesis of PP.

As clinical findings revealed the critical metabolomic changes in PP, in order to further explore the impact of DEMs on the disease, we further constructed a PP model by feeding newborn mice with danazol (26). Danazol, a derivative of 17α-ethinyl testosterone, can increase hypothalamic GnRH expression and accelerate the maturation of the HPG axis, thus advancing puberty. This danazol treatment method shortens the time of vaginal opening and estrous cycle, which are consistent with normal puberty in animals. The danazol-treated animal represents a unique model for analysis of the onset of puberty (27). In this study, the results showed that danazol advanced the time of vaginal opening in rats, increased the indexes of the uterus and ovary organs. Meanwhile, the mature corpora lutea and follicles were able to be observed in ovaries. These supported the successful establishment of the PP rat model in our studies. In the PP animal model, we employed metabolomic analysis and identified 180 differential metabolites in feces. These DEMs mainly include fatty acids, steroids and their derivatives, endogenous metabolites, glycerophospholipids and phenols. Analysis of KEGG results from animal samples showed that these DEM-enriched pathways overlapped with the clinical DEMs (biosynthesis of unsaturated fatty acids, glycerolipid metabolism, pyrimidine metabolism, steroid hormone biosynthesis, ovarian steroidogenesis) to a large extent, which were strongly associated with disease progression (steroid hormone biosynthesis, ovarian steroidogenesis, progesterone-mediated oocyte maturation, GnRH signaling pathway).

Progestogens, estrogens, androgens, and corticosteroids all belong to steroid hormones; they are critical in regulating human growth, neurobehavioral development, and reproductive behaviors. During perinatal development, steroid hormones act on the central nervous system (CNS) to organize neural circuits that remain relatively dormant at this stage; while in puberty, steroid hormone activation stimulates neural circuits, activates adult reproductive physiology and behavior, and further promotes CNS organization and behavior programming (28, 29). Accumulation studies have proven that EDC exposure disrupts sex steroid hormones in children and adolescents, causing endocrine disruption and other disorders. In a cross-sectional study involving 3,392 subjects aged 6–19 years in the USA, the data indicated gender- and age-specific negative associations of fluoride in plasma and water with sex steroid hormones of total testosterone, E_2_, and sex hormone-binding globulin (30). Another similar study also reported that EDC exposure [bisphenol A (BPA), bisphenol S (BPS), methylparaben (MeP), parabens, and phthalate metabolites] are negatively associated with E_2_, total testosterone, and free androgen index while positively associated with sex hormone–binding globulin (SHBG) in 6–19-year-old children, particularly in pubertal children. Those associations were stronger in pubertal children (31). In our previous study, we observed steroid hormone changes in the blood samples of PP girls, including those of hydrocortisone, 11-deoxycortisol, corticosterone, deoxycorticosterone, and pregnenolone, and also found that EDC exposure may affect the metabolism of steroid hormones in the body (32). In a similar study, steroid hormone biosynthesis was also identified as an enriched pathway for DEMs of CPP girls (33). Oocyte maturation and ovarian follicle development are key events in sexual maturity. The data suggest that steroidogenesis and lipid reserves are responsible for oocyte maturation and ovarian follicle development (34). In our study, the increased levels of steroid hormones were found in PP group girls; progesterone, pregnanediol, and 17a-hydroxypregnenolone were also identified as steroidal DEMs in PP rats. These all suggest the significant role of steroid hormones and steroidogenesis in PP.

Among these identified DEMs in PP rats, two DEMs overlap with clinical DEMs: thymine and inosine. The limited overlap DEMs between patient and animal model may be due to the inability of the animal model to fully simulate disease progression in a clinical setting, resulting in differences in disease-related metabolites. In turn, the overlapped DEMs from animal model do further support the clinical metabolomic findings. Moreover, except for a higher fold change of expression in clinical samples, clinical correlation analysis revealed a significant negative correlation between thymine levels and E_2_ in girls. Therefore, we investigated the impact of thymine on PP in the animal model, and the results showed that thymine supplementation delayed PP. Thymine, a pyrimidine base, is one of the four nucleobases found in DNA; the deoxyribonucleotide it is composed of is called a deoxythymidine nucleotide (dTMP). Within humans, thymine participates in a series of enzymatic reactions and is essential to life. Pyrimidine nucleotide synthesis is primarily initiated by the condensation of CO_2_ and glutamine to generate carbamoyl phosphate under catalysis by CPS-2; carbamoyl phosphate is further catalyzed by aspartate transcarbamoylase (ATCase) and dihydroorotase to synthesize dihydroorotate, which further activates with phosphoribosyl pyrophosphate (PRPP) to form orotidine-5′-monophosphate (OMP) and is catalyzed by OMP decarboxylase to generate the uridine nucleotide (UMP), and dTMP is generated by the methylation of UMP (35–37). In human cells, the activities of CPS-II, ATCase, and dihydrolactase are contained in a trifunctional protein CAD; deregulation of CAD-related pathways or CAD mutations cause cancer, neurological disorders, and inherited metabolic diseases (36). In our studies, except for the delayed time of vaginal opening in PP rats, thymine supplementation also changed CAD and CPS-II expression, indicating that thymine addition altered pyrimidine metabolism and affected PP progression. Previous studies also revealed the protective potential of thymine in human disorders. In polycystic ovary syndrome (PCOS) patients with insulin resistance, thymine was found to be increased in follicular fluid compared to that in non-insulin-resistant PCOS patients (38). In addition, tryptophan and thymine supplementation was able to inhibit urothelial carcinogenesis and potentiate intravesical Bacillus Calmette–Guérin in the treatment of bladder cancer (17).

Moreover, this study also performed next-generation sequencing in PP model rats and identified 425 DEGs, of which the typical downregulated genes included Plpp1, Hsd3b7, Scarb1, Pld3, Acadsb, and Gnai1 and of which the upregulated genes included Faslg, Cyp2u1, Pik3r3, Pla2g12a, Irf7, and Osbpl5. The direct association of these DEGs and PP is rare, but many reports provide potential clues between them. Scarb1 is critical for lipid synthesis and metabolism; its encoded protein is the plasma membrane receptor of high-density lipoprotein cholesterol (HDL), and the encoded protein can mediate the transfer between cholesterol and HDL (39). A study identified 16 unique variants of Gnai1 from 24 individuals, wherein the affected individuals suffered from severe neurodevelopmental disorders, including global developmental delay (40). Pik3r3 is a regulatory part of PI3K; dysregulated PI3K/Akt/mTOR in cervical cancer has been proved, and their inhibitors have been developed to treat cervical cancer (41). Some of these DEG-involved pathways were associated with PP, such as ovarian steroidogenesis, progesterone-mediated oocyte maturation, glycerolipid metabolism, fatty acid biosynthesis/degradation, arachidonic acid metabolism, and GnRH signaling pathway. In particular, the correlation heat map indicated tight connections of DEMs and DEGs; KEGG pathway analysis revealed 74 overlapping pathways in metabolomics and transcriptomics. The concordance between the metabolomic and transcriptomic results suggests that these DEMs and DEGs are able to form a tight DEM–DEG KEGG pathway regulatory network and are thus involved in the pathogenesis of PP.

Although some meaningful findings are obtained based on clinical and animal studies, there remain some shortcomings in this study. A further larger clinical sample size and multicenter study are recommended to adequately support the clinical findings, and boys with PP also need to be included. It may be that the animal model did not fully simulate the mechanism of precocious puberty, which resulted in a small number of overlaps between clinical and animal DEMs. For the validated thymine, its action mechanism has not been investigated comprehensively. More in-depth studies are needed to validate our findings in the future.

In summary, by metabolomic and transcriptomic analysis of PP girls and rat models, we identified 24 DEMs in clinical samples and 180 DEMs as well as 425 DEGs in animal samples. KEGG pathway analysis demonstrated that these DEMs and DEGs were enriched in PP-associated pathways, including fatty acid synthesis, glycerolipid metabolism, pyrimidine metabolism, steroid hormone biosynthesis, ovarian steroidogenesis, progesterone-mediated oocyte maturation, and GnRH signaling pathway. Additionally, a large overlap of the KEGG pathways enriched for DEMs and DEGs was found, thus forming a tight DEM–DEG KEGG pathway regulatory network. For the DEMs overlapping in clinical and animal samples, we further validate the impact of thymine on PP and found that thymine supplementation delayed the time of vaginal opening and development of PP in model rats. The findings of the present study may provide new strategies for PP treatment.

## Data availability statement

The original contributions presented in the study are included in the article/**Supplementary Material**. The data presented in the study are deposited in the NCBI repository, accession number PRJNA1071721. Further inquiries can be directed to the corresponding author.

## Ethics statement

The studies involving humans were approved by the Ethics Committee of Zhuji City Hospital of Traditional Chinese Medicine. The studies were conducted in accordance with the local legislation and institutional requirements. Written informed consent for participation in this study was provided by the participants’ legal guardians/next of kin. The animal study was approved by the Animal Experimentation Ethics Committee of Zhejiang Eyong Pharmaceutical Research and Development Center. The study was conducted in accordance with the local legislation and institutional requirements. Written informed consent was obtained from the individual(s), and minor(s)’ legal guardian/next of kin, for the publication of any potentially identifiable images or data included in this article.

## Author contributions

FZ: Conceptualization, Writing – original draft, Writing – review & editing. JM: Data curation, Writing – original draft. ZJ: Data curation, Writing – original draft. LZ: Data curation, Writing – review & editing. XL: Data curation, Writing – review & editing.
