# Supplementary material for: Multi-omic analysis of precocious puberty girls: pathway changes and metabolite validation
Source: Front Endocrinol (Lausanne). 2024 Feb 29;15:1285666. doi: 10.3389/fendo.2024.1285666 (PMC10937432; doi:10.3389/fendo.2024.1285666)
Supplement: Supplementary file 1 [file Table_1.docx]

**Supplementary Table 1** Differential metabolites identified from faeces of PP rats

| No. | **Metabolite** | **Formula** | **KEGG** | **P value** | **log_2_FC** | **VIP** |
| --- | --- | --- | --- | --- | --- | --- |
| 1 | Triethylamine | C6H15N | C14691 | 0.0001 | -0.99 | 1.83 |
| 2 | m-Cresol | C7H8O | C01467 | 0.0022 | 1.92 | 1.60 |
| 3 | L-3-Cyanoalanine | C4H6N2O2 | C02512 | 0.0407 | 1.12 | 1.30 |
| 4 | Glutarate semialdehyde | C5H8O3 | C03273 | 0.0266 | 1.02 | 1.25 |
| 5 | 4-Methylbenzaldehyde | C8H8O | C06758 | 0.0075 | 1.33 | 1.41 |
| 6 | Imidazoleacetic acid | C5H6N2O2 | C02835 | 0.0063 | 2.19 | 1.47 |
| 7 | Triacetate lactone | C6H6O3 | C02752 | 0.0032 | -2.44 | 1.79 |
| 8 | Thymine | C5H6N2O2 | C00178 | 0.0472 | -1.23 | 1.21 |
| 9 | (R)-5,6-Dihydrothymine | C5H8N2O2 | C21028 | 0.0417 | -0.27 | 1.18 |
| 10 | 2-Ketohexanoic acid | C6H10O3 | C00902 | 0.0008 | -0.98 | 1.64 |
| 11 | Heptanoic acid | C7H14O2 | C17714 | 0.0004 | -0.88 | 1.71 |
| 12 | Glutaric acid | C5H8O4 | C00489 | 0.0103 | 2.44 | 1.50 |
| 13 | Creatine | C4H9N3O2 | C00300 | 0.0000 | 4.14 | 1.96 |
| 14 | L-Leucine | C6H13NO2 | C00123 | 0.0151 | 1.62 | 1.49 |
| 15 | L-Asparagine | C4H8N2O3 | C00152 | 0.0462 | 1.85 | 1.31 |
| 16 | 4-Methylbenzoic acid | C8H8O2 | C01454 | 0.0138 | -0.89 | 1.58 |
| 17 | 4-Methoxybenzaldehyde | C8H8O2 | C10761 | 0.0038 | 2.64 | 1.61 |
| 18 | Quinolin-2-ol | C9H7NO | C06338 | 0.0008 | -1.45 | 1.68 |
| 19 | Coumarin | C9H6O2 | C05851 | 0.0063 | 3.81 | 1.52 |
| 20 | L-Lysine | C6H14N2O2 | C00047 | 0.0174 | 0.98 | 1.47 |
| 21 | L-Methionine | C5H11NO2S | C00073 | 0.0009 | -1.81 | 1.71 |
| 22 | Xanthine | C5H4N4O2 | C00385 | 0.0202 | -0.77 | 1.42 |
| 23 | FAPy-adenine | C5H7N5O | C06502 | 0.0466 | -1.05 | 1.21 |
| 24 | 2,3-Butanediol | C4H10O2S2 | C00265 | 0.0351 | 1.09 | 1.36 |
| 25 | Pelargonic acid | C9H18O2 | C01601 | 0.0116 | -0.3 | 1.48 |
| 26 | 1,5-Naphthalenediamine | C10H10N2 | C19463 | 0.0091 | 1 | 1.45 |
| 27 | Pimelic acid | C7H12O4 | C02656 | 0.0075 | -2.08 | 1.48 |
| 28 | Tryptamine | C10H12N2 | C00398 | 0.0242 | 1.06 | 1.37 |
| 29 | 3D-3,5_4-Trihydroxycyclohexane-1,2-dione | C6H8O5 | C04287 | 0.0372 | 1.04 | 1.19 |
| 30 | 2-Dehydro-3-deoxy-L-rhamnonate | C6H10O5 | C03979 | 0.0080 | 0.75 | 1.56 |
| 31 | Indole-3-carboxylic acid | C9H7NO2 | C19837 | 0.0166 | 2.31 | 1.35 |
| 32 | Tryptophanol | C10H11NO | C00955 | 0.0000 | -1.74 | 1.93 |
| 33 | L-Carnitine | C7H15NO3 | C00318 | 0.0002 | -1.91 | 1.74 |
| 34 | 2-Deoxystreptamine | C6H14N2O3 | C02627 | 0.0014 | 1.85 | 1.65 |
| 35 | Eugenol | C10H12O2 | C10453 | 0.0235 | 0.95 | 1.42 |
| 36 | Quinolinic acid | C7H5NO4 | C03722 | 0.0062 | 1.7 | 1.60 |
| 37 | D-synephrine | C9H13NO2 | C01869 | 0.0315 | 0.42 | 1.25 |
| 38 | Isopyridoxal | C8H9NO3 | C06051 | 0.0000 | 2.87 | 1.98 |
| 39 | 2-Keto-6-acetamidocaproate | C8H13NO4 | C05548 | 0.0204 | -1.34 | 1.26 |
| 40 | Pyridoxine | C8H11NO3 | C00314 | 0.0243 | -1.36 | 1.29 |
| 41 | Iminoarginine | C6H12N4O2 | C21026 | 0.0379 | 1.01 | 1.31 |
| 42 | (2S,5S)-trans-Carboxymethylproline | C7H11NO4 | C17366 | 0.0001 | -2.33 | 1.85 |
| 43 | Formiminoglutamic acid | C6H10N2O4 | C00439 | 0.0247 | -1.08 | 1.46 |
| 44 | Serotonin | C10H12N2O | C00780 | 0.0085 | 0.7 | 1.40 |
| 45 | Herniarin | C10H8O3 | C09268 | 0.0141 | -1.07 | 1.50 |
| 46 | 2,4-Dinitrotoluene | C7H6N2O4 | C11006 | 0.0191 | -1.31 | 1.48 |
| 47 | Glycylleucine | C8H16N2O3 | C02155 | 0.0056 | 1.26 | 1.43 |
| 48 | 7,8-Diaminononanoate | C9H20N2O2 | C01037 | 0.0003 | 1.09 | 1.72 |
| 49 | 4-Hydroxy-2-quinolinecarboxylic acid | C10H7NO3 | C01717 | 0.0146 | 3.45 | 1.40 |
| 50 | Diaminopimelic acid | C7H14N2O4 | C00666 | 0.0359 | -0.74 | 1.44 |
| 51 | L-Kynurenine | C10H12N2O3 | C00328 | 0.0094 | -1.58 | 1.41 |
| 52 | Sinapyl alcohol | C11H14O4 | C02325 | 0.0037 | 1.74 | 1.53 |
| 53 | gamma-Glutamyl-beta-aminopropiononitrile | C8H13N3O3 | C06114 | 0.0309 | -0.11 | 1.21 |
| 54 | Thiabendazole | C10H7N3S | C07131 | 0.0050 | 1.72 | 1.52 |
| 55 | Asymmetric dimethylarginine | C8H18N4O2 | C03626 | 0.0109 | 1.44 | 1.57 |
| 56 | beta-Cubebene | C15H24 | C09648 | 0.0000 | -3.19 | 1.86 |
| 57 | Indolelactic acid | C11H11NO3 | C02043 | 0.0379 | -1.32 | 1.36 |
| 58 | Pyrimidodiazepine | C9H11N5O2 | C02587 | 0.0237 | -1.02 | 1.32 |
| 59 | 2-trans,6-trans-Farnesal | C15H24O | C03461 | 0.0474 | -1.83 | 1.21 |
| 60 | N-Acetyl-D-glucosamine | C8H15NO6 | C00140 | 0.0001 | -0.67 | 1.82 |
| 61 | Apiole | C12H14O4 | C10429 | 0.0441 | 2.81 | 1.30 |
| 62 | Butyryl-L-carnitine | C11H21NO4 | C02862 | 0.0023 | -1.2 | 1.65 |
| 63 | Alantolactone | C15H20O2 | C09289 | 0.0017 | -2.64 | 1.69 |
| 64 | Confertifolin | C15H22O2 | C09376 | 0.0141 | 0.76 | 1.40 |
| 65 | Pyridoxal 5'-phosphate | C8H10NO6P | C00018 | 0.0000 | -1.17 | 1.91 |
| 66 | Deoxyadenosine | C10H13N5O3 | C00559 | 0.0161 | 2.78 | 1.29 |
| 67 | 16-Oxopalmitate | C16H30O3 | C19614 | 0.0033 | -2.44 | 1.57 |
| 68 | Saccharopine | C11H20N2O6 | C00449 | 0.0374 | 1.12 | 1.37 |
| 69 | Phenylacetylglutamine | C13H16N2O4 | C04148 | 0.0403 | -3.35 | 1.38 |
| 70 | Norlinolenic acid | C17H28O2 | C16344 | 0.0001 | 1.5 | 1.82 |
| 71 | Androsterone | C19H30O2 | C00523 | 0.0160 | -0.97 | 1.48 |
| 72 | Neocembrene | C20H32 | C09140 | 0.0156 | -1.12 | 1.39 |
| 73 | Nandrolone | C18H26O2 | C07254 | 0.0003 | -2.43 | 1.81 |
| 74 | Alpha-Linolenic acid | C18H30O2 | C06427 | 0.0166 | -1.84 | 1.55 |
| 75 | 3-Ketosphingosine | C18H35NO2 | C06121 | 0.0026 | 1.5 | 1.63 |
| 76 | N-Glucosylnicotinate | C12H16NO7 | C03003 | 0.0127 | -1.87 | 1.54 |
| 77 | 4'-Oxonebramine | C12H24N4O5 | C21258 | 0.0196 | 1.1 | 1.30 |
| 78 | N-(L-Arginino)succinate | C10H18N4O6 | C03406 | 0.0162 | -2.46 | 1.38 |
| 79 | Etiocholanolone | C19H30O2 | C04373 | 0.0003 | -1.56 | 1.85 |
| 80 | 13(S)-HpOTrE | C18H30O4 | C04785 | 0.0068 | -1.48 | 1.53 |
| 81 | 9,10-12,13-Diepoxyoctadecanoate | C18H32O4 | C14836 | 0.0239 | -1.42 | 1.48 |
| 82 | 9,10-DHOME | C18H34O4 | C14828 | 0.0021 | -0.95 | 1.69 |
| 83 | 9,10-Dihydroxystearate | C18H36O4 | C19622 | 0.0409 | -1.01 | 1.23 |
| 84 | (R)-10-Hydroxystearate | C18H36O3 | C03195 | 0.0178 | 2.09 | 1.35 |
| 85 | Diosmetin | C16H12O6 | C10038 | 0.0117 | -0.96 | 1.54 |
| 86 | 8-HETE | C20H32O3 | C14776 | 0.0035 | -2.52 | 1.59 |
| 87 | Dihydrocapsaicin | C18H29NO3 | C16952 | 0.0010 | 1.57 | 1.59 |
| 88 | 10-Nitrolinoleic acid | C18H31NO4 | C13800 | 0.0233 | -1.21 | 1.49 |
| 89 | Eicosadienoic acid | C20H36O2 | C16525 | 0.0095 | -1.82 | 1.50 |
| 90 | Dicyclomine | C19H35NO2 | C06951 | 0.0021 | -3.63 | 1.60 |
| 91 | Aflatoxin B1 | C17H12O6 | C06800 | 0.0049 | -2.23 | 1.62 |
| 92 | Levonorgestrel | C21H28O2 | C08153 | 0.0358 | 1.28 | 1.28 |
| 93 | Progesterone | C21H30O2 | C00410 | 0.0045 | 4.53 | 1.71 |
| 94 | Menthyl pyrrolidone carboxylate | C16H30O6 | C03962 | 0.0263 | 0.77 | 1.22 |
| 95 | 19(R)-HETE | C20H32O3 | C14749 | 0.0001 | -3.56 | 1.87 |
| 96 | Pregnanediol | C21H36O2 | C05484 | 0.0043 | -2.63 | 1.53 |
| 97 | Citalopram | C20H21FN2O | C07572 | 0.0025 | -2.94 | 1.60 |
| 98 | 17alpha,21-Dihydroxypregnenolone | C21H32O4 | C05487 | 0.0000 | -3.68 | 1.97 |
| 99 | 17a-Hydroxypregnenolone | C21H32O3 | C05138 | 0.0004 | 6.53 | 1.90 |
| 100 | 12-Keto-leukotriene B4 | C20H30O4 | C05949 | 0.0021 | -3.21 | 1.68 |
| 101 | Prostaglandin D2 | C20H32O5 | C00696 | 0.0358 | 1.53 | 1.19 |
| 102 | Prostaglandin C1 | C20H32O4 | C04686 | 0.0203 | 1.18 | 1.28 |
| 103 | 5,6-DHET | C20H34O4 | C14772 | 0.0110 | 2.04 | 1.50 |
| 104 | 14,15-DiHETrE | C20H34O4 | C14775 | 0.0077 | 2.93 | 1.65 |
| 105 | Cortexolone | C21H30O4 | C05488 | 0.0024 | -1.73 | 1.75 |
| 106 | N-Acetylmuramoyl-Ala | C14H24N2O9 | C02999 | 0.0033 | -1.89 | 1.62 |
| 107 | Bufadienolide | C24H34O2 | C16921 | 0.0388 | 1.77 | 1.31 |
| 108 | (4Z,7Z,10Z,13Z,16Z,19Z)-Docosahexaenoic acid ethyl ester | C24H36O2 | C16185 | 0.0000 | 2.41 | 1.93 |
| 109 | 11-Dehydro-thromboxane B2 | C20H32O6 | C05964 | 0.0000 | -2.45 | 1.99 |
| 110 | Bufalin | C24H34O4 | C16922 | 0.0015 | -2.75 | 1.86 |
| 111 | Adenosine 5'-phosphate disodium | C10H12N5O7P. 2Na |  | 0.0322 | 1.06 | 1.20 |
| 112 | Gamma-Tocotrienol | C28H42O2 | C14155 | 0.0345 | 0.62 | 1.13 |
| 113 | 17a,20a-Dihydroxycholesterol | C27H46O3 | C05499 | 0.0228 | 1.5 | 1.28 |
| 114 | 4alpha-Methylcholesta-8-en-3beta-ol | C28H48O | C05110 | 0.0001 | -1.77 | 1.83 |
| 115 | Lovastatin | C24H36O5 | C07074 | 0.0369 | -0.48 | 1.26 |
| 116 | 14alpha-Hydroxy-5beta-cholest-7-ene-3,6-dione | C27H42O3 | C16509 | 0.0001 | 3.44 | 1.83 |
| 117 | 7a,12a-Dihydroxy-5b-cholestan-3-one | C27H46O3 | C05453 | 0.0121 | 0.7 | 1.36 |
| 118 | Alpha-Tocotrienol | C29H44O2 | C14153 | 0.0109 | 1.56 | 1.49 |
| 119 | Taraxerol | C30H50O | C08637 | 0.0027 | 1.83 | 1.56 |
| 120 | N1,N8-Bis(4-coumaroyl)spermidine | C25H31N3O4 | C21169 | 0.0096 | -1.4 | 1.47 |
| 121 | Arabidiol | C30H52O2 | C19828 | 0.0029 | 0.53 | 1.54 |
| 122 | alpha-Elemolic acid | C30H48O3 | C08623 | 0.0041 | 1.88 | 1.55 |
| 123 | 20-Hydroxyecdysone | C27H44O7 | C02633 | 0.0386 | -1.66 | 1.22 |
| 124 | Cucurbitacin F | C30H46O7 | C08798 | 0.0473 | -0.47 | 1.31 |
| 125 | Apiin | C26H28O14 | C04858 | 0.0004 | -1.59 | 1.74 |
| 126 | Betaine | C5H11NO2 | C00719 | 0.0008 | 1.26 | 1.61 |
| 127 | Nicotinic acid | C6H5NO2 | C00253 | 0.0000 | -6.08 | 1.85 |
| 128 | 4-Methylcatechol | C7H8O2 | C06730 | 0.0083 | -1.37 | 1.39 |
| 129 | L-Isoleucine | C6H13NO2 | C00407 | 0.0031 | 1.67 | 1.53 |
| 130 | (3R)-beta-Leucine | C6H13NO2 | C02486 | 0.0050 | 1.62 | 1.48 |
| 131 | L-Malic acid | C4H6O5 | C00149 | 0.0224 | -1.23 | 1.33 |
| 132 | Hypoxanthine | C5H4N4O | C00262 | 0.0132 | 1.82 | 1.35 |
| 133 | Ribitol | C5H12O5 | C00474 | 0.0010 | 2.19 | 1.59 |
| 134 | p-Hydroxyphenylacetic acid | C8H8O3 | C00642 | 0.0443 | -1.61 | 1.18 |
| 135 | 2,5-Dihydroxybenzoate | C7H6O4 | C00628 | 0.0010 | -2.09 | 1.59 |
| 136 | L-Histidine | C6H9N3O2 | C00135 | 0.0090 | -0.57 | 1.41 |
| 137 | Phenyllactate | C9H10O3 | C05607 | 0.0003 | -3.83 | 1.67 |
| 138 | Phthalic acid | C8H6O4 | C01606 | 0.0001 | -7.64 | 1.80 |
| 139 | 3,4-Dihydroxybenzeneacetic acid | C8H8O4 | C01161 | 0.0455 | -1.05 | 1.26 |
| 140 | 1H-Indole-3-acetamide | C10H10N2O | C02693 | 0.0031 | 1.42 | 1.44 |
| 141 | N-Acetyl-L-aspartic acid | C6H9NO5 | C01042 | 0.0344 | -2.98 | 1.31 |
| 142 | D-Gulono-1,4-lactone | C6H10O6 | C05410 | 0.0001 | -5.12 | 1.81 |
| 143 | Nicotinuric acid | C8H8N2O3 | C05380 | 0.0460 | -0.69 | 1.14 |
| 144 | Caffeate | C9H8O4 | C01197 | 0.0491 | -1.03 | 1.11 |
| 145 | Alpha-D-Glucose | C6H12O6 | C00267 | 0.0262 | 1.33 | 1.28 |
| 146 | N-Acetylglutamic acid | C7H11NO5 | C00624 | 0.0195 | -1.03 | 1.33 |
| 147 | Quinate | C7H12O6 | C00296 | 0.0023 | -2.48 | 1.49 |
| 148 | Citric acid | C6H8O7 | C00158 | 0.0002 | -2.58 | 1.68 |
| 149 | D-Glucuronic Acid | C6H10O7 | C00191 | 0.0011 | -0.87 | 1.58 |
| 150 | Gluconic acid | C6H12O7 | C00257 | 0.0045 | -2.18 | 1.35 |
| 151 | Syringic acid | C9H10O5 | C10833 | 0.0216 | 2.78 | 1.29 |
| 152 | L-Tryptophan | C11H12N2O2 | C00078 | 0.0031 | -2.11 | 1.44 |
| 153 | N-Acetyl-L-phenylalanine | C11H13NO3 | C03519 | 0.0454 | -2.08 | 1.26 |
| 154 | 3-(3,4-Dihydroxy-5-methoxy)-2-propenoic acid | C10H10O5 | C05619 | 0.0157 | -1.07 | 1.40 |
| 155 | 6-Acetyl-D-glucose | C8H14O7 | C02655 | 0.0346 | -3.49 | 1.35 |
| 156 | Citrinin | C13H14O5 | C16765 | 0.0064 | 1.86 | 1.41 |
| 157 | Galactosylglycerol | C9H18O8 | C05401 | 0.0227 | -1.84 | 1.32 |
| 158 | (2S)-Liquiritigenin | C15H12O4 | C09762 | 0.0227 | -3.29 | 1.22 |
| 159 | beta-D-Fructose 6-phosphate | C6H13O9P | C05345 | 0.0167 | 2.51 | 1.30 |
| 160 | Inosine | C10H12N4O5 | C00294 | 0.0003 | -2.13 | 1.68 |
| 161 | Naringenin | C15H12O5 | C00509 | 0.0079 | -2.04 | 1.51 |
| 162 | [8]-Shogaol | C17H24O3 | C10494 | 0.0015 | -2.94 | 1.60 |
| 163 | Taxifolin | C15H12O7 | C01617 | 0.0285 | -1.49 | 1.34 |
| 164 | 13S-hydroxyoctadecadienoic acid | C18H32O3 | C14762 | 0.0036 | 2.38 | 1.44 |
| 165 | 9,10-Epoxyoctadecenoic acid | C18H32O3 | C14825 | 0.0342 | 1.04 | 1.17 |
| 166 | Hispidulin | C16H12O6 | C10058 | 0.0021 | -2.5 | 1.63 |
| 167 | all-trans-Retinoic acid | C20H28O2 | C00777 | 0.0006 | 3.2 | 1.65 |
| 168 | EPA (d5) | C20H30O2 | C06428 | 0.0004 | 3.14 | 1.63 |
| 169 | Arachidonic acid | C20H32O2 | C00219 | 0.0016 | -1.18 | 1.55 |
| 170 | 9(S)-HPODE | C18H32O4 | C14827 | 0.0078 | -1.85 | 1.41 |
| 171 | 5-KETE | C20H30O3 | C14732 | 0.0059 | -0.82 | 1.37 |
| 172 | Gentiobiose | C12H22O11 | C08240 | 0.0469 | -1.45 | 1.15 |
| 173 | 11-Dehydrocorticosterone | C21H28O4 | C05490 | 0.0202 | -1.46 | 1.26 |
| 174 | Docosapentaenoic acid (22n-3) | C22H34O2 | C16513 | 0.0007 | 1.55 | 1.57 |
| 175 | Sucrose | C12H22O11 | C00089 | 0.0045 | -1.7 | 1.50 |
| 176 | alpha,alpha-Trehalose | C12H22O11 | C01083 | 0.0384 | -1.79 | 1.18 |
| 177 | Cellobiose | C12H22O11 | C00185 | 0.0051 | -2.77 | 1.42 |
| 178 | Perindopril | C19H32N2O5 | C07706 | 0.0113 | 5.76 | 1.50 |
| 179 | Chenodeoxycholic acid | C24H40O4 | C02528 | 0.0000 | 2.56 | 1.73 |
| 180 | Ergocalciferol | C28H44O | C05441 | 0.0213 | 1.84 | 1.26 |
